# A Novel method for the identification and quantification of weight faltering

**DOI:** 10.1002/ajpa.24217

**Published:** 2021-01-01

**Authors:** Daniel J. Naumenko, James Dykes, G. Kesler O'Connor, Zofia Stanley, Nabeel Affara, Andrew M. Doel, Saikou Drammeh, David B. Dunger, Abdoulie Faal, Ken K. Ong, Fatou Sosseh, Andrew M. Prentice, Sophie E. Moore, Robin M. Bernstein

**Affiliations:** ^1^ Growth and Development Lab, Department of Anthropology University of Colorado Boulder Boulder Colorado USA; ^2^ Health and Society Program, Institute of Behavioral Science University of Colorado Boulder Boulder Colorado USA; ^3^ Department of Applied Mathematics University of Colorado Boulder Boulder Colorado USA; ^4^ Department of Pathology University of Cambridge Cambridge UK; ^5^ Department of Women and Children's Health King's College London London UK; ^6^ MRC Unit The Gambia at London School of Hygiene and Tropical Medicine Banjul Gambia; ^7^ Department of Paediatrics University of Cambridge School of Clinical Medicine Cambridge UK; ^8^ Institute of Metabolic Science Cambridge Biomedical Campus Cambridge UK; ^9^ MRC Epidemiology Unit University of Cambridge School of Clinical Medicine Cambridge UK

**Keywords:** growth faltering, infancy, stunting, the Gambia, wasting

## Abstract

**Objective:**

We describe a new method for identifying and quantifying the magnitude and rate of short‐term weight faltering episodes, and assess how (a) these episodes relate to broader growth outcomes, and (b) different data collection intervals influence the quantification of weight faltering.

**Materials and methods:**

We apply this method to longitudinal growth data collected every other day across the first year of life in Gambian infants (*n* = 124, males = 65, females = 59). Weight faltering episodes are identified from velocity peaks and troughs. Rate of weight loss and regain, maximum weight loss, and duration of each episode were calculated. We systematically reduced our dataset to mimic various potential measurement intervals, to assess how these intervals affect the ability to derive information about short‐term weight faltering episodes. We fit linear models to test whether metrics associated with growth faltering were associated with growth outcomes at 1 year, and generalized additive mixed models to determine whether different collection intervals influence episode identification and metrics.

**Results:**

Three hundred weight faltering episodes from 119 individuals were identified. The number and magnitude of episodes negatively impacted growth outcomes at 1 year. As data collection interval increases, weight faltering episodes are missed and the duration of episodes is overestimated, resulting in the rate of weight loss and regain being underestimated.

**Conclusions:**

This method identifies and quantifies short‐term weight faltering episodes, that are in turn negatively associated with growth outcomes. This approach offers a tool for investigators interested in understanding how short‐term weight faltering relates to longer‐term outcomes.

## 1 INTRODUCTION

Determining whether an individual's growth is due to natural/normal variation or is indicative of adverse conditions, and pinpointing when growth perturbations begin (Victora et al., 2010), are areas of continued research and debate. It is standard and near‐universal practice among health care workers to monitor growth during infancy and childhood to pick up on potential disturbances to a child's growth trajectory as potential indicators of challenges to health (de Onis & Branca, 2016). Growth standards, such as World Health Organization (WHO)‐guided thresholds for wasting and stunting (i.e., below‐2SDs from the WHO Child Growth Standards median for weight‐for‐height and height‐for‐age z‐scores, respectively [WHO Multicentre Growth Reference Study Group, 2006]) are internationally accepted (de Onis et al., 2013; WHO, 2008). While metrics such as wasting and stunting may indicate when an individual is not growing normally relative to a reference population, they dichotomize the inherently continuous process of growth faltering (de Onis & Branca, 2016) and have only been able to resolve the initiation of the state of growth faltering as happening in utero or the first 1000 days of life (Christian et al., 2013; de Onis et al., 2013; Dewey & Huffman, 2009). Thus, the appropriate method for determining when a growth perturbation is initiated and subsequently resolved, and what combination of factors caused the perturbation, remains unresolved.

Several approaches can identify growth faltering or loss of growth potential, but each have drawbacks that preclude the study of the proximate mechanisms of acute faltering events. First, WHO Growth Reference (WHO Multicentre Growth Reference Study Group, 2006) cutoffs indicate small size or stature relative to the reference, with a height‐for‐age (HAZ), weight‐for‐age (WAZ), and weight‐for‐height (WFH) < −2 *SD* as the standard cutoff, as noted above (Alderman & Headey, 2018). Although these cutoffs are often used to indicate faltering in a given anthropometric dimension at a given age, they are limited in utility as an individual who falls at or below these cutoffs may have started out small, meaning they are tracking their appropriate trajectory. This static approach only measures attained growth at a given time point, and encompasses neither change over time or minimizes variation in growth trajectories among individuals (Eveleth & Tanner, 1976; Lampl et al., 2015). Second, raw growth cutoffs use a predefined gain in weight or height below which is indicative of faltering (Martorell & Shekar, 1994). However, this approach has no independent, robust basis for cutoff selection; individual researchers must arbitrarily decide how little gain or how much loss is indicative of faltering. Third, centile crossing indicates an individual deviating from their growth trajectory; here, faltering is defined as when an individual crosses down more than two major (i.e., 5th, 10th, 25th, 50th, 75th, 90th, 95th) centiles (Wright, 2000). Although this method is more dynamic in its ability to look at change over time, counting centiles is both noncontinuous and imprecise due to the uneven spacing of the centiles. Fourth, z‐score deviations and conditional anthropometry are usually applied to height (Argyle, 2003), with a negative change in z‐score indicative of faltering (Cole, 1993; Healy, 1974; Shrimpton et al., 2001; Victora et al., 2010; Waterlow et al., 1977). Although the generation of conditional z‐scores requires normally distributed anthropometric data (Waterlow et al., 1977) or transformative procedures which presuppose otherwise undefined “normal” growth (Cole, 1993), they have been applied to shorter collection intervals (Cole, 1995; Wright et al., 2006).

Though the latter two methods of assessing growth faltering permit detection of an individual deviating from their growth trajectory, there exists a gap in our ability to quantify those deviations in a standardized and precise way. A new approach is needed to link the deviation from one's trajectory with the proximate mechanisms that caused the individual to falter, then further link this to explicit adverse conditions (particularly during the first 1000 days of life) with later growth and health outcomes. Growth faltering has two facets: (a) the state of faltering, related to *potential* growth comprised of genetic potential plus factors that constrain how much one can growth at a given time, resulting in a given outcome for age (e.g., a specific z‐score); and (b) faltering episodes, or short‐term growth deficits resulting from various factors and which cumulatively result in the state of growth faltering (e.g., an individual ending up smaller than they “should” have given their initial trajectory). Growth faltering both as a process and as an outcome has been linked to a myriad potential negative impacts increased morbidity and disease risk (Black et al., 2008; Hoffman, 2014; Olofin et al., 2013), reduced stature (Stein et al., 2010), and increased risk of obesity from subsequent catch‐up growth (Ong et al., 2000; Ong et al., 2015). To better understand how the process of growth faltering leads to different growth and health outcomes, we need to be able to characterize the specific aspects of faltering, such as timing, magnitude, and rate.

Here, we propose a novel method for identifying weight growth faltering episodes and quantifying their magnitude and rate, and demonstrate its utility and application using longitudinal weight data from a cohort of Gambian infants, designed to explore growth in the first 1000 days in fine detail (Moore et al., 2020). Our primary objective is to apply this method to identify, for each individual infant growth trajectory across the first year of life, weight‐faltering episodes that are mathematically defined based on changes in growth velocity, and to determine what effect the magnitude and rate of these episodes have on growth outcomes at 1 year of age. Tracking growth velocity, specifically changes in growth velocity, has proven to be a sensitive method of monitoring changes in growth due to pathology (Tanner, 1990). To maximize the utility of this method for future study design and implementation, our second objective is to explore the relationship between measurement collection interval and faltering metrics identified, and specifically to assess the extent of information lost when measurements are collected over broader intervals.

## 2 MATERIALS AND METHODS

### 2.1 Study subjects and site

Data for this study were obtained from the Hormonal and Epigenetic Regulators of Growth (HERO‐G) cohort, recruited from a rural subsistence farming population in the West Kiang region of The Gambia. The full study protocol has been published elsewhere (Moore et al., 2020). Infants from the cohort were visited by a trained field assistant every alternate day from 9 to 365 days of post‐natal life. At each visit, a panel of anthropometrics were collected. The current analysis focuses on weight, which was recorded on handheld tablets in triplicate to the nearest 10 g using a Seca 336 digital weighing scale. Some models also include outcomes calculated based on infant length (HAZ, WHZ). Infant crown‐heel length was also recorded in triplicate to the nearest 0.1 cm, using a Seca 417 length board. Field assistants received comprehensive training in anthropometric measurement at the start of the study; regular standardization exercises were performed to measure inter‐observer variation (Moore et al., 2020). Data capture software entry was designed such that each anthropometric measurement was taken once, after which the screen would refresh and each measurement would be taken again, following collection of the full panel of anthropometric measures, to help reduce intra‐observer bias. Infants were weighed naked where possible; in some cases, light twine bracelets or necklaces, worn at all times throughout the study, remained on an infant even after clothing was removed. Mothers refrained from breastfeeding during measurement and were asked to not feed their infant prior to measurement. A total of 124 infants (male = 65 with 142–177 measurements each, female = 59 with 140–175 measurements each) were included in this study from the original cohort (*N* = 238) based on these individuals having near‐complete weight growth curves; specifically, HERO‐G infants were included in this analysis if they had fewer than five missing consecutive weight measurements across the first year of life. The average of all three replicate weight measures was used when the coefficient of variation (CV) of the triplicates was below 5% (99.5% of observations). If CV was greater than 5%, and one triplicate was at least one whole number above or below the remaining two triplicates, then that triplicate was dropped and the average of the remaining two was used instead. Intraobserver technical error of measurement (TEM) was calculated for each anthropometrist (*N* = 21) and their collected data using one set of triplicate measurements from each study subject from each month (12 monthly triplicate measurements per individual), following the equation $\mathrm{TEM}=\sqrt{\;\frac{\sum D^{2}}{2N}\;}$, where D equals each replicate deviation, and N equals the number of subjects that fieldworker measured (Ulijaszek & Kerr, 1999). See “Statistics” section below for information regarding application of intra‐observer TEM.

The HERO‐G study was approved by the joint Gambia Government / MRC Unit The Gambia Ethics Committee (Project number SCC1313v3), and the University of Colorado Boulder Institutional Review Board (protocol number 13–0441). Prior to the start of the study, community approval was obtained from each participating village. Participating women were given the option of either reading the consent form on their own or having it read to them by a trained field assistant in their language. After given an opportunity to consider their involvement and talk to family members, written, informed consent was obtained from each participating mother.

### 2.2 Faltering episode identification and quantification: Method description

**FIGURE 1:**
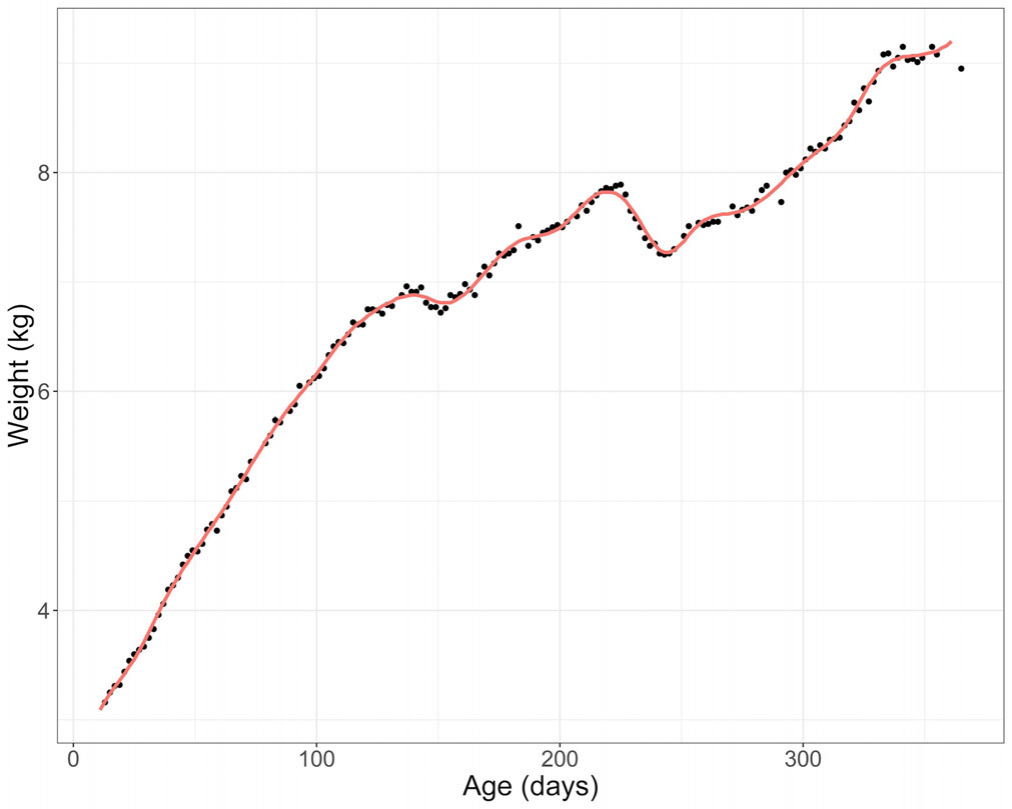
A single individual's raw weight curve plotted over the first year of life. Red line indicating the chosen 25‐knot cubic spline. Spline curve is sensitive to larger fluctuations in weight while smoothing out day‐to‐day natural variation in weight measurements

**FIGURE 2:**
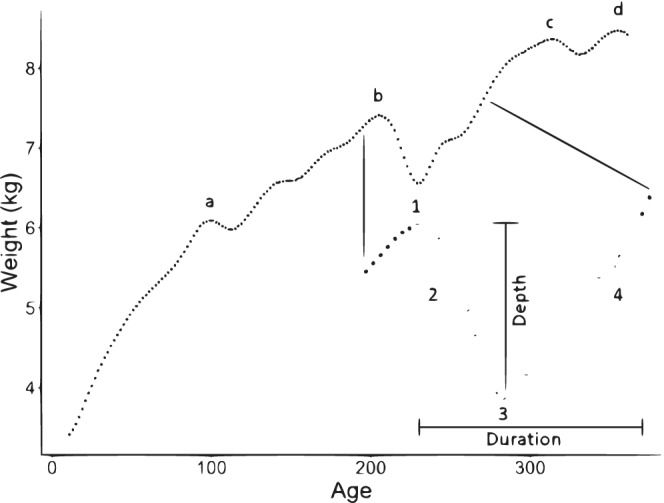
Demonstration of method application to a single individual. Smoothed weight is plotted over the first year of life, and each faltering episode identified. Letters indicate each faltering episode (four total falter episodes). Numbers and colors indicate faltering episode stages: 1—Initiation (+): transition from positive to negative growth rate, at which the first derivative is equal to zero; 2—dip (▽): loss of weight following episode initiation; 3—depth maximum (X): transition from negative to positive growth rate, at which the first derivative is equal to zero; 4—rebound (□): gain of weight following maximum depth until weight at time of episode Initiation is re‐attained

The following approach was applied to individual weight growth curves, which identified faltering episodes, deconstructed each into component stages, and quantified their magnitude. For each individual, a linear interpolation was performed to impute missing data, given the limited amount of missing data. A 25‐knot cubic spline was then fitted to the resulting weight curve to smooth out minor day‐to‐day fluctuations, potentially due to the intake and excretion of breas tmilk, water, and non‐breas tmilk food, while still retaining the overall shape of the curve (Figure 1). This knot was chosen by fitting different knots (range: 5–50) in iterative fashion to individual growth trajectories, and visually identifying which knot best followed the shape of the trajectory without overfitting day‐to‐day shifts. The first derivative was then calculated across the splined weight curve, and peaks and troughs identified where the first derivative was equal to zero using the *find_peaks* function (Aphalo, 2018; see R code in Supplementary Materials). Stages of individual faltering episodes (Figure 2:1–4) were defined as:

1. Initiation: when the first derivative transitioned from positive to negative (i.e., a peak).
2. Dip: the subsequent period of weight loss.
3. Depth maximum: when the first derivative transitioned from negative to positive (i.e., a trough).
4. Rebound: the period of weight gain following depth maximum, continuing until the individual re‐attains the weight at which the episode was initiated. The last weight measurement equal to or less than the weight at Initiation of the episode is the final measurement in the rebound stage, at which point the episode terminates. If the measurement immediately following depth maximum is higher (i.e., in weight) than the measurement at Initiation, the episode terminates at the depth maximum.

**FIGURE 3:**
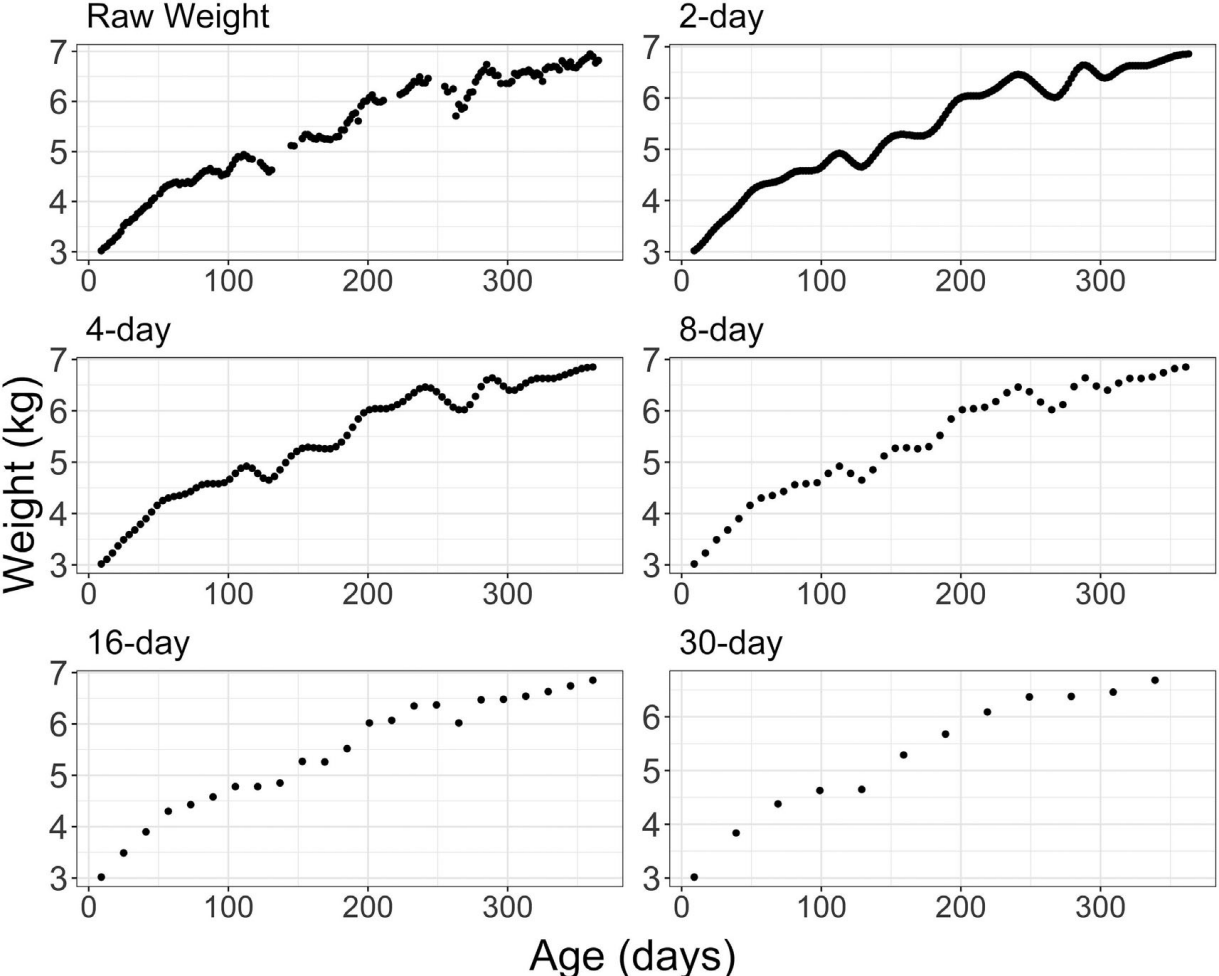
Demonstration of method application to different data collection intervals. The observed data are plotted in the upper left panel. Remaining panels indicate the smoothed spline curve at the 2‐, 4‐, 8‐, 16‐, and 32‐day interval, respectively

Using this method, a range of metrics can be calculated from each identified faltering episode (Figure 2a–d). Individual episodes and their stages can be quantified in terms of their duration (length of time from Initiation to the end of rebound), rate (i.e., of loss and regain), and depth (maximum amount of weight lost during the episode). Number of faltering events initiated, the average depth of the episodes, and the proportion of time spent within faltering episodes (encompassing initiation, dip, depth maximum, and rebound) across the first year were calculated and compared between individuals. Rate of weight loss during the dip period for each faltering episode was calculated from Initiation to depth maximum, and the rate of weight gain during the rebound period was calculated from depth maximum to the last observation equal to or less than the Initiation of the given faltering episode. Each rate is expressed as grams/day, for comparison between the different collection intervals. Neither rate was calculated for a given faltering episode if (a) the rebound stage did not start before the end of data collection for the particular subject (day 365), or (b) if the weight measurement following the faltering episode's depth maximum was higher than the weight at Initiation. In this second case, the duration of the rebound period cannot be calculated, either. This is not an issue for the raw data used in this study but becomes relevant when looking at data mimicking longer collection intervals (see below). As an example, the individual shown in Figure 3 shows a faltering episode Initiation ~250 days. At anthropometric collection intervals up to and including once weekly, the true rebound of this particular faltering episode is successfully identified. When measured twice monthly or monthly, however, the rebound of this episode is missed.

### 2.3 Statistics

To evaluate measurement error in a longitudinal and study‐specific way, mean intra‐observer TEM across fieldworkers in this study (0.005 kg) was calculated as $\sqrt{\frac{\sum D^{2}}{2N}}$, with D being the deviation two given replicates and N being the total number of subjects that fieldworker measured. This was then used to calculate the amount of weight loss (>14 g) within a given episode in excess of which we could be 95% confident was not due to measurement error alone, using the equation $2*\sqrt{{\left(\mathrm{TEM}\right)}^{2}+{\left(\mathrm{TEM}\right)}^{2}}$ (Ulijaszek & Kerr, 1999). This provides a diagnostic tool to filter out episodes by estimating the proportion of difference between two measures, in this case the Initiation and depth maximum of a given episode, beyond which might be attributed to measurement error alone. Episodes with a depth in excess of 14 g were retained for analysis. Given the frequency of data collection, a potential issue arises regarding intra‐daily micro‐changes in weight, such as those due to urination, where identified episodes may be due to non‐tissue changes in weight. We reran all analyses excluding episodes with a depth in excess of 74 g, obtained by addition of the initial 14 g threshold plus 60 g representing the upper range of weight of a full (60 ml) bladder of a 9‐month old infant (Guerra et al., 2014). We report both sets of results, and refer to results based on the 14 g threshold in subsequent text unless otherwise stated.

We fit linear models to determine if derived faltering metrics predict broad changes to attained growth trajectory across the first year. Weight and height were converted into WAZ, HAZ, and WFH according to WHO references (WHO Multicentre Growth Reference Study Group, 2006) at our first (day 9 of postnatal life) and last (12 months) measurements used in the current analysis. Twelve month z‐scores were set as outcomes and faltering metrics (number of falters, average falter depth, average rate of dip and rebound and their interaction, sex, and baseline z‐score) as predictors. Model fit was assessed for normality and heteroscedasticity of residuals, and multicollinearity. Terms were dropped in sequential fashion if any variance inflation factor (VIF) exceeded five (Sheather, 2009) and AIC was reduced by at least two, until all terms fell below five. To determine whether episodic effects are more characterized by acute or chronic weight loss, we fit a generalized additive mixed model (GAMM) with depth as the outcome and dip and rebound durations as smoothed predictors using the MGCV package in R (R Development Core Team, 2015; Wood, 2006, 2019).

To test whether increasing the collection interval length, and thus decreasing anthropometric data resolution, alters the identification and quantification of growth faltering using this method, we subset our data to mimic anthropometry collected at intervals of 4, 8, 16, and 32 days. These are serial doublings of our original collection interval, roughly approximate to measurements collected twice weekly, weekly, twice monthly, and monthly. We then applied the same faltering method protocol to each collection interval, quantifying the number of faltering episodes and their metrics, and averaging each metric across the collection period for each study subject. GAMMs were fitted with the eight individual‐level faltering metrics (count, depth, proportion, episode duration, dip duration, rebound duration, dip rate, and rebound rate) as outcomes, with sex and collection interval fit as linear and smooth terms, respectively, and subject ID as a random effect. Episode count was fit with a Poisson distribution while the other seven metrics were fit with Gaussian distributions. All descriptive statistics are presented as means ± standard deviation. Identification and quantification of faltering episodes, and all subsequent statistical analyses, were performed in Rv3.6.1. (R Development Core Team, 2015).

## 3 RESULTS

We identified 300 weight faltering episodes from 119 individuals (2.52 ± 1.14, range: 1–6, Table 1). Five individuals had no detected weight faltering episodes; each of these individuals was born in the dry season, and four were conceived in the wet season. On average, infants spent ~9% of their first year of life within weight faltering episodes, lost 170 g per episode at a rate of 8.9 g/day, and regained that lost weight at a rate of 9.9 g/day. These episodes lasted an average of 1 month, with considerable variation in total episode duration (range: 16–170 days). Further, the dip and rebound stages both exhibited wide variation in duration (dip:10–48 days; rebound:5–148 days), despite having similar averages (dip:18.32 days; rebound:14.59 days). When using the stricter 74 g threshold for inclusion, our results show that infants spent ~10% of their first year of life within weight faltering episodes, lost 254 g per episode at a rate of 12.7 g/day, and regained that lost weight at a rate of 13.3 g/day, remaining generally consistent with the results using the 14 g threshold for inclusion, with higher values reflecting the higher weight cutoff.

**TABLE 1:** Faltering episode metrics calculated using each simulated interval

		Interval (days)									
		2		4		8		16		32	
**Inclusion threshold (g)**		**14 g**	**74 g**	**14 g**	**74 g**	**14 g**	**74 g**	**14 g**	**74 g**	**14 g**	**74 g**
**Subjects**	N	119 (96%)	100 (81%)	118 (95%)	100 (81%)	115 (93%)	95 (77%)	105 (85%)	84 (68%)	58 (47%)	42 (34%)
**Count**	Mean	2.52	1.83	2.45	1.75	2.30	1.59	1.86	1.45	1.17	1.12
	*SD*	1.14	0.93	1.15	0.93	1.08	0.84	0.84	0.70	0.38	0.33
	Min	1.00	1	1.00	1	1.00	1	1.00	1	1.00	1
	Max	6.00	5	6.00	5	5.00	5	5.00	4	2.00	2
**Depth**	Mean	169.90	253.83	166.12	247.09	160.61	250.04	175.78	252.23	193.45	256.07
**(g)**	*SD*	146.09	184.84	142.19	185.47	141.23	184.82	154.93	180.86	184.20	189.09
	Min	20.00	80.00	20.00	80.00	20.00	80.00	20.00	80.00	20.00	80.00
	Max	1115.00	1115.00	1115.00	1115.00	1100.00	1100.00	980.00	980.00	850.00	850.00
**Proportion**	Mean	0.09	0.10	0.11	0.12	0.16	0.15	0.22	0.19	0.22	0.19
**(% first year)**	*SD*	0.05	0.04	0.07	0.06	0.12	0.09	0.13	0.12	0.08	0.04
	Min	0.04	0.05	0.03	0.04	0.02	0.02	0.04	0.04	0.16	0.16
	Max	0.47	0.26	0.37	0.35	0.57	0.46	0.53	0.48	0.41	0.25
**Dip duration**	Mean	18.32	21.81	18.57	22.2	19.23	22.88	22.43	25.81	35.95	37.5
**17.29**	*SD*	6.12	6.81	6.92	7.54	6.83	7.96	8.36	12.01	15.37	17.29
	Min	10.00	11.00	8.00	12.00	8.00	8.00	16.00	16.00	30.00	30.00
	Max	48.00	48.00	52.00	52.00	48.00	48.00	48.00	80.00	90.00	90.00
**Rebound duration**	Mean	14.59	16.22	21.73	22.01	41.11	30.51	58.72	46.04	45.00	33.33
**(days)**	*SD*	16.82	10.25	25.41	18.98	43.20	33.51	50.06	44.15	30.00	10.00
	Min	4.00	4.00	4.00	4.00	8.00	8.00	16.00	16.00	30.00	16.00
	Max	148.00	70.00	128.00	100.00	192.00	160.00	176.00	160.00	120.00	60.00
**Episode duration**	Mean	32.90	38.08	40.00	44.82	60.44	53.47	80.41	70.67	80.00	70.00
**(days)**	*SD*	19.34	13.99	26.13	21.75	42.05	33.32	47.21	42.08	29.54	15.00
	Min	16.00	18.00	12.00	16.00	16.00	16.00	16.00	16.00	60.00	60.00
	Max	170.00	96.00	136.00	128.00	208.00	168.00	192.00	176.00	150.00	90.00
**Dip rate**	Mean	−8.93	−12.72	−8.03	−10.98	−7.68	−11.05	−7.61	−10.36	−5.69	−7.52
**(g/day)**	*SD*	10.50	16.41	4.97	6.32	5.14	6.35	5.45	6.01	5.57	5.85
	Min	−1.25	−3.54	−1.00	−3.86	−1.25	−3.33	−1.25	−2.5	−0.67	−1.67
	Max	−108.77	−162.08	−32.66	−39.38	−36.39	−36.39	−31.67	−31.67	−28.33	−28.33
**Rebound rate**	Mean	9.94	13.03	8.01	11.28	7.47	9.47	6.96	8.57	4.33	5.70
**(g/day)**	*SD*	6.14	8.29	6.00	7.53	6.16	7.62	7.82	8.89	4.10	3.82
	Min	1.67	0	0.48	0	0.11	0	0.25	0	0.33	1.00
	Max	44.55	44.55	50.56	50.56	47.66	47.66	40.63	40.62	12.00	12.00

*Note*: Values represent subjects (*N*) with identifiable faltering episodes after adjustment for TEM (depth > 14 g) or TEM and non‐tissue weight change (depth > 74 g) at each time interval. Included is the percentage of individuals with identified episodes out of all included subjects (*N* = 124). Mean, *SD*, minimum, and maximum are calculated for each metric by interval.

Attained growth models (WAZ, HAZ, WFH at 12 months as the outcome) each followed a similar pattern in relation to faltering episode metrics (Table 2). After adjustment for baseline z‐score, the interaction of dip and rebound rates, and subsequently dip rate itself, were removed from all models due to nonsignificance and high VIF, respectively. The remaining faltering metrics (count, depth, and rebound rate) explained the most variance in WAZ (*R* ^2^_ajd_ = 0.27), followed by HAZ (*R* ^2^_ajd_ = 0.18) and WFH (*R* ^2^_ajd_ = 0.08). WAZ was inversely associated with number of faltering episodes (β = −0.14). Greater depth (i.e., more weight lost) was associated with lower WAZ (β = −1.77) and HAZ (β = −2.31), but not WFH. Increased rebound rate was associated with higher WAZ (β = 52.81), HAZ (β = 61.28), and WFH (β = 48.51). Sex did not emerge as a significant predictor in any model. dip duration, but not rebound duration, was a significant predictor of depth (*R* ^2^_adj_ = 0.43, F_(4.37)_ = 25.47, *p* < 0.0001), with increasing dip duration corresponding with greater weight lost. Models fit with data from the 74 g inclusion threshold were consistent with those fit with the lower 14 g threshold, except that average depth had a weaker effect, R^2^_adj_ increased, and AIC decreased in all three models (Table S1).

**TABLE 2:** Outputs for models of z score outcome against faltering metrics

Model		Full model statistics	Model terms	β (95% CI)	t‐stat, *p*‐value
1	WAZ_endline_ ~ sex + WAZ_baseline_ + # of falters + depth + rebound rate	*r* ^2^_adj_ = 0.27 F_(5,97)_ = 8.42 ***p* < 0.0001** AIC = 238.35	Intercept	−0.59 (−1.05, −0.12)	t = −2.5, ***p* = 0.014**
			Sex	−0.25 (−0.55, 0.05)	t = −1.67, *p* = 0.1
			WAZ at birth	0.47 (0.28, 0.5)	t = 5.03, ***p* < 0.0001**
			# of falters	−0.14 (−0.27, −0.01)	t = −2.12, ***p* = 0.037**
			Average Depth	−1.77 (−3.33, −0.20)	t = −2.23, ***p* = 0.028**
			Average rebound rate	52.81 (15.88, 89.75)	t = 2.84, ***p* < 0.01**
2	HAZ_endline_ ~ Sex + HAZ_baseline_ + # of falters + depth + rebound rate	*r* ^2^_adj_ = 0.18 F_(5,97)_ = 5.31 ***p* < 0.001** AIC = 251.24	Intercept	−0.98 (−1.44, −0.52)	t = −4.24, ***p* < 0.0001**
			Sex	−0.04 (−0.36, 0.28)	t = −0.25, *p* = 0.80
			HAZ at birth	0.24 (0.12, 0.36)	t = 3.93, ***p* < 0.001**
			# of falters	−0.009 (−0.15, 0.13)	t = −0.13, *p* = 0.90
			Average depth (kg)	−2.31 (−3.4, −0.64)	t = 02.75, ***p* < 0.01**
			Average rebound rate	61.28 (22.43, 100.13)	t = 3.13, ***p* < 0.01**
3	WFH_endline_ ~ Sex + WFH_baseline_ + # of falters + depth + rebound rate	*r* ^2^_adj_ = 0.08 F_(5,95)_ = 2.69 ***p* = 0.03** AIC = 275.11	Intercept	−0.59 (−1.14, −0.04)	t = −2.14, ***p* = 0.035**
			Sex	−0.31 (−0.68, 0.06)	t = −1.65, *p* = 0.1
			WFH at Birth	0.11 (0.01, 0.21)	t = 2.08, ***p* = 0.04**
			# of falters	−0.15 (−0.31, 0.01)	t = −1.82, *p* = 0.07
			Average depth (kg)	−1.39 (−3.31, 0.53)	t = −1.43, *p* = 0.16
			Average rebound rate	48.51 (2.68, 94.34)	t = 2.1, ***p* = 0.038**

*Note*: Depth and rebound rate units in kilograms. Current models resulted from initial removal of nonsignificant dip rate x rebound rate interaction term, and removal of dip rate due to high collinearity. Female is the baseline sex. Each model checked for normality of residuals using a Shapiro–Wilk test, and for homoscedasticity of residuals using a score test for nonconstant error variance. Multicollinearity was assessed using variance inflation factor.

Simulated collection interval significantly predicted all derived faltering metrics irrespective of sex (Table 1). As simulated collection interval increased from 2 to 32 days, the total number of identifiable weight faltering episodes dropped, as did the total number of subjects with at least one identifiable episode (Table 1). Average, as well as minimum and maximum, number of identified episodes dropped significantly as collection interval increased (*R* ^2^_adj_ = 0.15, F_(1)_ = 46.84, *p* < 0.0001). Episode depth decreased from 2‐ to 8‐day intervals, then increased up to the 32‐day interval, indicating that longer collection intervals can overestimate depth, though minimal variation in depth overall was explained by interval (*R* ^2^_adj_ = 0.012, F_(1)_ = 4.86, *p* = 0.028). Episode depth remained roughly consistent in value across all collection intervals when using the 74 g inclusion criterion (Table 1). Proportion of time spent in episodes increased up to the 16‐day interval then leveled off (*R* ^2^_adj_ = 0.21, F_(2.46)_ = 42.05, *p* < 0.0001). Average proportions remained relatively low across intervals (average: 9%–22%), though some individuals spent a considerable proportion of their first year within faltering episodes (maximum: 37%–57%).

These proportion changes are reflected in the duration and rate metrics. Dip and rebound duration both increased with greater collection interval from ~2 weeks to over 1 month (dip: *R* ^2^_adj_ = 0.286, interval: F_(2.55)_ = 117, *p* < 0.0001; rebound: *R* ^2^_adj_ = 0.176, interval: F_(2.56)_ = 32.02, *p* < 0.0001). Episode duration, being a by‐product of these durations, followed the same pattern (*R* ^2^_adj_ = 0.214, F_(2.46)_ = 42.24, *p* < 0.0001). Dip and rebound rate both trended towards zero as interval increased, indicating a drift towards underestimation with increasing collection interval. Collection interval significantly influenced both rates (dip rate, interval: F_(1)_ = 23.84, *p* < 0.001; rebound rate, interval: F_(1.86)_ = 21.15, *p* < 0.0001), though they explained little variance in outcome (dip rate: *R* ^2^_adj_ = 0.02; rebound rate: *R* ^2^_adj_ = 0.043).

## 4 DISCUSSION

We present a new method and framework for identifying and quantifying short‐term weight faltering episodes. This approach, and the ability to quantify aspects of individual episodes, can provide the context to link acute conditions to the broader state of faltering from an individual's potential growth trajectory. Faltering metrics significantly predicted relative attained growth at 1 year after accounting for size at 1 week, suggesting that they are relevant for predicting infant growth outcomes. As these are weight faltering episodes, it is not surprising that they would have the strongest effect on and explain the most variance in WAZ. Their relationship to HAZ is noteworthy, though they explain only ~20% of variance in HAZ at 1‐year (Table 2). While the number of episodes did not impact linear growth, average depth had a dramatic effect: for every 1 kg of average depth, there was a − 2.31 drop in HAZ at 1 year. That the mean depth of episodes was ~170 g, and ~ 95% of episodes had a depth < 814 g, suggests such deep episodes are uncommon (Table 1). Taken together with the positive association between dip duration and depth, this suggests that weight faltering negatively effects linear growth through pathways that can include frequent, low magnitude episodes and/or infrequent, high magnitude episodes. Further, the rate of rebound is significant in all models, following either inclusion criterion, except for WFH at the stricter threshold (Table S1), indicating this period as a critical window for the determination of growth outcomes. This finding builds on prior work in this population that demonstrated repeated rounds of wasting increases the risk of stunting (Schoenbuchner et al., 2019). The positive association between rebound rate and each growth outcome suggests that understanding the dynamics of weight growth faltering are critical for understanding the mechanisms underlying these outcomes, and their continued prevalence across much of the world (Shrimpton et al., 2001; Victora et al., 2010).

Simulated collection interval significantly influenced all faltering metrics in infants. In all cases, increasing the interval across which anthropometrics were collected led to loss of information, especially at the monthly interval. As collection intervals increase, faltering episodes were missed, the amount of actual weight lost deviated from that recorded in the 2‐day interval, and dip and rebound rates were underestimated, making some episodes seem less severe than they actually were. Though collecting more frequent anthropometric data can be logistically challenging, twice monthly and monthly intervals in this population appear to be insufficient to reveal the episodic nature of short‐term weight loss and regain (Figure 3), as they underestimate the severity (i.e., rate and depth) of the rate at which it proceeds, and increasingly miss episodes to the point that many individuals appear to experience none at all. Because both rate metrics are prone to underestimation, the shortest possible collection interval should be used if either are to be captured and their contribution assessed. The relationship between lower magnitude weight faltering episodes and longer‐term growth outcomes is unclear, and the cost in time and resources of more frequent measurement, versus the risk of missing smaller episodes should be carefully considered within the context of overall monitoring goals.

Although we applied this method to a data set retrospectively for the purposes of method development, we propose that it can be implemented in real‐time in field research and clinical settings to identify the initiation of faltering episodes, providing critical information regarding a weight faltering episode's magnitude, and allowing for the timely deployment of (a) targeted intervention measures to rectify the faltering episode, and/or (b) additional strategic growth data collection to follow the response to intervention in real time. For example, a researcher could monitor the individual illustrated in Figure 3 at all but the monthly interval and still identify weight faltering episodes using the approach presented here. When an Initiation is noted (such as the one shortly after 100 days), that individual would be flagged and the faltering episode followed up. Depending on the aims of the study that employs the method, follow up could call for evaluation by a community health worker and treatment if needed, a shift to a higher‐resolution collection interval, initiation of nutritional intervention, clinical evaluation, and so on. Future studies should be sure to consider potential non‐tissue changes in weight; a full bladder, recent feeding, or the presence of undergarments could inflate observed weight without affecting actual tissue weight, potentially resulting in false identification of faltering episodes. Overall, this method offers a standardized framework for exploring and responding to the dynamics of growth faltering across settings, and allowing a more nuanced understanding of the causes and consequences of short‐term weight loss/regain in infants and young children.

We suggest that this method complement centile crossing (Wright, 2000) and z‐score deviations (Argyle, 2003; Cole, 1995) approaches for analyzing growth faltering. Stunting and growth faltering remain global problems despite fervent and focused efforts to combat them (Shrimpton et al., 2001; Victora et al., 2010), and new approaches to the identification and analysis of faltering are needed. If the global issue of stunting is to be addressed, its dynamic causes must be identified and explored. From a mechanistic perspective, it is clear that repeated rounds of growth faltering (e.g., resulting from high morbidity, undernutrition, repeated seasonal pressures, or other adverse conditions) could potentially (a) deplete resources available to support growth (Stearns, 1989), and (b) signal a “high risk” environment to the developing individual, potentially impacting growth patterns and health in both the short‐ and long‐term (Bernstein et al., 2020; Gluckman et al., 2005). Close analysis of the dynamics of faltering episodes as presented here can help tease apart the causes and consequences of faltering, shed light on how those episodes contribute to the broader state of faltering from a “potential” trajectory identified by prior methods, and may provide valuable insight regarding the efficacy of interventions designed to improve growth and growth outcomes.

There are three main limitations to this study. First, the method cannot be applied to height measurements. This method assumes that cessation of weight growth, or weight loss, during this time period is abnormal, and thus indicative of deviation from a healthy growth trajectory. Height does not necessarily follow this same pattern. Two models of height growth, saltation, and stasis (Lampl et al., 1992) and mini‐growth spurts (Hermanussen, 1998; Hermanussen et al., 1998) both incorporate lack of growth (i.e., stasis) in height within the definition of normal growth, suggesting apparent reductions in height gain are normal and thus precluding the application of the method described here to height. Second, while we reran our analyses with a higher exclusion threshold based on bladder fullness and found consistent results with the lower threshold, the possibility remains that a small number of the weight faltering episodes we identified could have been due to additional non‐tissue weight differences that we did not account for. Third, this study focuses only on outcomes at the end of the first year of life. We therefore cannot yet fully link rebound to catch‐up growth across different stages of growth, given the range of potential catch‐up strategies. Catch‐up growth can occur by increasing growth rate above normal or extending the duration of growth (Tanner, 1981); potentially, the latter could involve individuals catching up through adolescence (Allal et al., 2004). As such, rebound as we have defined it here may not encompass catch‐up growth that involves an extension of the overall growth period, and should not be considered to imply catch‐up of growth potential. Thus, while rebound (as used here) may encompass certain types of catch‐up growth, we recommend pairing this method with an approach which tackles growth potential, such as z‐score deviations.

In summary, this study presents a novel method for identifying and quantifying weight growth faltering, and tests the impact of different collection intervals on the refinement of weight faltering metrics. Incorporating this method into future study designs will require tailoring data collection intervals to specific research questions, given the changes to timing and duration components of episode metrics that are associated with longer collection intervals. We hope that future applications of this method will allow greater insights into what causes weight growth faltering, how the body responds to challenges in the context of various intrinsic and extrinsic factors, and what the long‐term consequences for individual growth and health outcomes might be.

## AUTHOR CONTRIBUTIONS

**Daniel Naumenko:** Conceptualization; data curation; formal analysis; methodology; software; visualization; writing‐original draft; writing‐review and editing. **James Dykes:** Conceptualization; writing‐review and editing. **G. Kesler O'Connor:** Conceptualization; writing‐review and editing. **Zofia Stanley:** Conceptualization; writing‐review and editing. **Nabeel Affara:** Funding acquisition; methodology; project administration; writing‐review and editing. **Andrew Doel:** Investigation; project administration; writing‐review and editing. **Saikou Drammeh:** Investigation; writing‐review and editing. **David Dunger:** Funding acquisition; methodology; project administration; writing‐review and editing. **Abdoulie Faal:** Data curation; investigation; writing‐review and editing. **Ken Ong:** Funding acquisition; methodology; project administration; writing‐review and editing. **Fatou Sosseh:** Investigation; writing‐review and editing. **Andrew Prentice:** Funding acquisition; methodology; project administration; writing‐review and editing. **Sophie Moore:** Funding acquisition; methodology; project administration; writing‐review and editing. **Robin Bernstein:** Conceptualization; data curation; funding acquisition; methodology; project administration; supervision; writing‐original draft; writing‐review and editing.

## CONFLICT OF INTEREST

The authors declare there are no conflict of interest.

## Supporting information


**Appendix** S1: Supporting Information

Table S1

## Data Availability

The raw data supporting the conclusions of this manuscript are stored on the Open Science Framework (OSF), DOI 10.17605/OSF.IO/5ND3Y, and at the time of manuscript submission are available on request and subject to ethics review. These data will be made publicly available no later than July 1, 2021. Requests to access the datasets should be directed to the corresponding author.
