# Supplementary material for: A Novel method for the identification and quantification of weight faltering
Source: Am J Phys Anthropol. 2021 Jan 1;175(1):282–91. doi: 10.1002/ajpa.24217 (PMC8247282; doi:10.1002/ajpa.24217)
Supplement: Supplementary file 1 — Appendix S1: Supporting Information [file AJPA-175-282-s002.docx]

Faltering Manuscript Code Submission

Daniel J. Naumenko

5/4/2020

This code is a cleaned form of that used in the AJPA publication Naumenko et al. XXXX. A novel method for the identification and quantification of growth faltering. An example of episode metric quantification is used for the 2-day data. Simulation of 4-, 8-, 16-, and 30-day collection intervals is shown.

library(tidyverse)
library(zoo)
library(gridExtra)
library(knitr)
library(lme4)
library(MuMIn)

# create a row shift function
rowShift <- function(x, shiftLen = 1L) {
 r <- (1L + shiftLen):(length(x) + shiftLen)
 r[r<1] <- NA
 return(x[r])
}


# peak finding function from: https://stats.stackexchange.com/questions/22974/how-to-find-local-peaks-valleys-in-a-series-of-data
# the higher the 'm' argument is, the more stringent the peak finding function
# this function is now contained in the "ggpmisc" package

find_peaks <- function (x, m = 3){
 # x is a sequential vector
 # x finds local maxima
 # -x finds local minima
 # m is the number of points to either side of a given point which must be below to identify as a maxima
 shape <- diff(sign(diff(x, na.pad = FALSE)))
 pks <- sapply(which(shape < 0), FUN = function(i){
 z <- i - m + 1
 z <- ifelse(z > 0, z, 1)
 w <- i + m + 1
 w <- ifelse(w < length(x), w, length(x))
 if(all(x[c(z : i, (i + 2) : w)] <= x[i + 1])) return(i + 1) else return(numeric(0))
 })
 pks <- unlist(pks)
 pks
}

# set working directory

# read in raw weight data

df_weight <- read_csv("./herog_weight.csv")

df_falter <- df_weight %>%
 # interpolate missing data
 # cSubjectID = unique subject identifier
 group_by(cSubjectID) %>%
 # Weight = replicate average following Naumenko et al. inclusion criteria
 # use linear interpolation to fill missing raw data
 # fit 25 knot spline to smooth out day to day minor weight fluctuations. Knot may need adjusting,
 # depending on the amount of data collected. Interpolation should be limited, as faltering
 # episodes can be short.
 mutate(interp_2day = na.approx(Weight, na.rm = FALSE),
 spline_2day = smooth.spline(x = Age, y = interp_2day, keep.data = TRUE, nknots = 25)$y)

# two day will just be the data as is

day_2 <- seq(from = 9, to = 365, by = 2)

# 4-day

day_4 <- seq(from = 9, to = 365, by = 4)


# 8-day

day_8 <- seq(from = 9, to = 365, by = 8)


# 16-day

day_16 <- seq(from = 9, to = 365, by = 16)


# 30-day

day_30 <- seq(from = 9, to = 365, by = 30)


# reduced data to mimic alternative collection intervals
df_falter_spline <- df_falter %>%
 mutate(weight_2 = Weight_spline_25knot,
 weight_4 = ifelse(Age %in% day_4, spline_2day, NA),
 weight_8 = ifelse(Age %in% day_8,spline_2day, NA),
 weight_16 = ifelse(Age %in% day_16, spline_2day, NA),
 weight_30 = ifelse(Age %in% day_30, spline_2day, NA))

# create data frame for 2-day

df_falter_spline_2day <- df_falter_spline %>%
 filter(Age %in% day_2) %>%
 select(cSubjectID:interp_2day, weight_2) %>%
 group_by(cSubjectID) %>%
 mutate(ID_obs = as.numeric(rownames(.)))


# create data frame for 4-day

df_falter_spline_4day <- df_falter_spline %>%
 filter(Age %in% day_4) %>%
 select(cSubjectID:interp_2day, weight_4) %>%
 group_by(cSubjectID) %>%
 mutate(ID_obs = as.numeric(rownames(.)))


# create data frame for 8-day
df_falter_spline_8day <- df_falter_spline %>%
 filter(Age %in% day_8) %>%
 select(cSubjectID:interp_2day, weight_8) %>%
 group_by(cSubjectID) %>%
 mutate(ID_obs = as.numeric(rownames(.)))


# create data frame for 16-day
df_falter_spline_16day <- df_falter_spline %>%
 filter(Age %in% day_16) %>%
 select(cSubjectID:interp_2day, weight_16) %>%
 group_by(cSubjectID) %>%
 mutate(ID_obs = as.numeric(rownames(.)))

# create data frame for 30-day
df_falter_spline_30day <- df_falter_spline %>%
 filter(Age %in% day_30) %>%
 select(cSubjectID:interp_2day, weight_30) %>%
 group_by(cSubjectID) %>%
 mutate(ID_obs = as.numeric(rownames(.)))

# 2-day

df_falter_peak_id_2day <- df_falter_spline_2day %>%
 group_by(cSubjectID) %>%
 # label peaks as Falter Initiation, and troughs as "Depth Maximum
 mutate(Deriv_maxmin_2day = ifelse(ID_obs %in% find_peaks(.$weight_2, m = 1), "Falter Initiation",
 ifelse(ID_obs %in% find_peaks(-(.$weight_2), m = 1), "Falter Depth Maximum", "Other Growth")))


# 4-day

df_falter_peak_id_4day <- df_falter_spline_4day %>%
 group_by(cSubjectID) %>%
 # label peaks as Falter Initiation, and troughs as "Depth Maximum
 mutate(Deriv_maxmin_4day = ifelse(ID_obs %in% find_peaks(.$weight_4, m = 1), "Falter Initiation",
 ifelse(ID_obs %in% find_peaks(-(.$weight_4), m = 1), "Falter Depth Maximum", "Other Growth")))


# 8-day

df_falter_peak_id_8day <- df_falter_spline_8day %>%
 group_by(cSubjectID) %>%
 # label peaks as Falter Initiation, and troughs as "Depth Maximum
 mutate(Deriv_maxmin_8day = ifelse(ID_obs %in% find_peaks(.$weight_8, m = 1), "Falter Initiation",
 ifelse(ID_obs %in% find_peaks(-(.$weight_8), m = 1), "Falter Depth Maximum", "Other Growth")))

# 16-day

df_falter_peak_id_16day <- df_falter_spline_16day %>%
 group_by(cSubjectID) %>%
 # label peaks as Falter Initiation, and troughs as "Depth Maximum
 mutate(Deriv_maxmin_16day = ifelse(ID_obs %in% find_peaks(.$weight_16, m = 1), "Falter Initiation",
 ifelse(ID_obs %in% find_peaks(-(.$weight_16), m = 1), "Falter Depth Maximum", "Other Growth")))


# 30-day

df_falter_peak_id_30day <- df_falter_spline_30day %>%
 group_by(cSubjectID) %>%
 # label peaks as Falter Initiation, and troughs as "Depth Maximum
 mutate(Deriv_maxmin_30day = ifelse(ID_obs %in% find_peaks(.$weight_30, m = 1), "Falter Initiation",
 ifelse(ID_obs %in% find_peaks(-(.$weight_30), m = 1), "Falter Depth Maximum", "Other Growth")))


# data frames were subsequently read out to csv files.
# The Dip period was filled in between each Initiation and subsequent Depth Maximum (or end of collection period)
# The Rebound period was filled in from Depth Maximum until weight at Initiation was reached.
# Termination was used to mark the last Rebound observation.

# read in data file from above code chunk, once Dip, Rebound, and Termination are filled in
df_falterstage_2day <- read.csv("./day2_falterstages.csv")


# 'Falter_Stage' replaces the 'Deriv_maxmin_#day' column, and contains the following character values:
# Initiation, Dip, Depth Maximum, Rebound, Termination, Other Growth

# calculate number of falters per individual


df_falterstage_2day %>%
 group_by(cSubjectID) %>%
 filter(Falter_Stage == "Initiation") %>%
 summarise(n_falter = n())


# calculate average depth of falters

df_falterstage_2day %>%
 group_by(cSubjectID) %>%
 filter(Falter_Stage %in% c("Initiation", "Depth Maximum")) %>%
 mutate(falter_depth = weight_2 - lag(weight_2, n = 1)) %>%
 filter(Falter_Stage == "Depth Maximum") %>%
 summarise(ave_falt_dept = mean(falter_depth))

# calculate proportion of observations


df_falterstage_2day %>%
 group_by(cSubjectID, Falter_Stage) %>%
 summarise(n = n()) %>%
 mutate(rel_freq = paste0(round(100 * n/sum(n), 0), "%")) %>%
 filter(Falter_Stage == "Other Growth")


# calculate dip and rebound rate


df_falterstage_2day %>%
 select(cSubjectID:Age, weight_2, Falter_Stage) %>%
 filter(Falter_Stage %in% c("Initiation", "Depth Maximum", "Termination")) %>%
 group_by(cSubjectID) %>%
 mutate(dip_rate = ((weight_2 - rowShift(weight_2, -1))/(Age - rowShift(Age, -1)))) %>%
 filter(Falter_Stage %in% c("Depth Maximum")) %>%
 summarise(ave_dip_rate = mean(dip_rate, na.rm = TRUE))

df_falterstage_2day %>%
 select(cSubjectID:Age, weight_2, Falter_Stage) %>%
 filter(Falter_Stage %in% c("Initiation", "Depth Maximum", "Termination")) %>%
 group_by(cSubjectID) %>%
 mutate(rebound_rate = ((weight_2 - rowShift(weight_2, -1))/(Age - rowShift(Age, -1)))) %>%
 filter(Falter_Stage %in% c("Termination")) %>%
 summarise(ave_rebound_rate = mean(rebound_rate, na.rm = TRUE))


# calculate duration of dip and rebound periods

df_rate_2day %>%
 select(cSubjectID:Age, weight_2, Falter_Stage) %>%
 filter(Falter_Stage %in% c("Initiation", "Depth Maximum", "Termination")) %>%
 group_by(cSubjectID) %>%
 mutate(dip_duration = Age - rowShift(Age, -1)) %>%
 filter(Falter_Stage %in% c("Depth Maximum")) %>%
 summarise(ave_dip_duration = mean(dip_duration, na.rm = TRUE))


df_rate_2day %>%
 select(cSubjectID:Age, weight_2, Falter_Stage) %>%
 filter(Falter_Stage %in% c("Initiation", "Depth Maximum", "Termination")) %>%
 group_by(cSubjectID) %>%
 mutate(rebound_duration = Age - rowShift(Age, -1)) %>%
 filter(Falter_Stage %in% c("Termination")) %>%
 summarise(ave_reb_duration = mean(rebound_duration, na.rm = TRUE))


# calculate duration of full faltering episode

df_rate_2day %>%
 select(cSubjectID:Age, weight_2, Falter_Stage) %>%
 filter(Falter_Stage %in% c("Initiation", "Depth Maximum", "Termination")) %>%
 group_by(cSubjectID) %>%
 mutate(dip_duration = Age - rowShift(Age, -1),
 episode_duration = dip_duration + rowShift(dip_duration, -1)) %>%
 filter(Falter_Stage %in% c("Termination")) %>%
 summarise(ave_episode_duration = mean(episode_duration, na.rm = TRUE))
