# Supplementary material for: A Novel method for the identification and quantification of weight faltering
Source: Am J Phys Anthropol. 2021 Jan 1;175(1):282–91. doi: 10.1002/ajpa.24217 (PMC8247282; doi:10.1002/ajpa.24217)
Supplement: Supplementary file 2 — Table S1 [file AJPA-175-282-s001.docx]

| **Model** | | **Full model statistics** | **Model terms** | **β (95% CI)** | **t-stat, p-value** |
| --- | --- | --- | --- | --- | --- |
| 1 | WAZ_endline_ ~ Sex + WAZ_baseline_ + # of Falters + Depth + Rebound Rate | r^2^ _adj_= 0.32  F_(5,81)_=9.12  **p<0.0001**  AIC=198.55 | Intercept | -0.43 (-0.92, 0.06) | t = -1.75, **p = 0.08** |
|  |  |  | Sex | -0.43 (-0.75, -0.1) | t = -2.59, **p=0.01** |
|  |  |  | WAZ at Birth | 0.49 (0.29, 0.70) | t = 4.87, **p<0.0001** |
|  |  |  | # of Falters | -0.26 (-0.42, -0.1) | t = -3.10, **p=0.003** |
|  |  |  | Average Depth | -0.48 (-1.62, 0.67) | t = -0.83, p=0.41 |
|  |  |  | Average Rebound Rate | 34.14 (8.79, 59.49) | t = 2.84, **p<0.01** |
| 2 | HAZ_endline_ ~ Sex + HAZ_baseline_ + # of Falters + Depth + Rebound Rate | r^2^ _adj_=0.23  F_(5,81)_=6.2  **p<0.0001**  AIC=204.09 | Intercept | -0.87 (-1.34, -0.4) | t = -3.7, **p < 0.001** |
|  |  |  | Sex | -0.26 (-0.6, 0.08) | t = -1.54, p=0.13 |
|  |  |  | HAZ at Birth | 0.28 (0.15, 0.41) | t = 4.4, **p<0.0001** |
|  |  |  | # of Falters | -0.10 (-0.27, 0.07) | t = -1.18, p=0.24 |
|  |  |  | Average Depth (kg) | -0.65 (-1.84, 0.54) | t = -1.09, p=0.28 |
|  |  |  | Average Rebound Rate | 44.21 (18.43, 69.99) | t = 3.41, **p<0.01** |
| 3 | WFH_endline_ ~ Sex + WFH_baseline_ + # of Falters + Depth + Rebound Rate | r^2^ _adj_=0.10  F_(5,79)_=2.79  **p=0.02**  AIC=230.21 | Intercept | -0.47 (1.04, 0.09) | t = -1.67, p = 0.098 |
|  |  |  | Sex | -0.39 (-0.8, 0.02) | t = -1.91, p=0.06 |
|  |  |  | WFH at Birth | 0.10 (-0.01, 0.22) | t = 1.76, p=0.08 |
|  |  |  | # of Falters | -0.24 (-0.45, -0.03) | t = -2.23, **p=0.03** |
|  |  |  | Average Depth (kg) | -0.37 (-1.79, 1.05) | t = -0.52, p=0.6 |
|  |  |  | Average Rebound Rate | 27.67 (-3.65, 60.0) | t = 1.76, p=0.08 |

Table S1: Outputs for models of z score outcome against faltering metrics, after removing all episodes with a depth below 74g to account for non-tissue micro-changes in weight . Depth and Rebound Rate units in kilograms. Current models resulted from initial removal of non-significant Dip Rate x Rebound Rate interaction term, and removal of Dip Rate due to high collinearity. Female is the baseline sex. Each model checked for normality of residuals using a Shapiro-Wilk test, and for homoscedasticity of residuals using a Score Test for Non-Constant Error Variance. Multicollinearity was assessed using variance inflation factor.
